# The Associations between Apolipoprotein E Gene Epsilon2/Epsilon3/Epsilon4 Polymorphisms and the Risk of Coronary Artery Disease in Patients with Type 2 Diabetes Mellitus

**DOI:** 10.3389/fphys.2017.01031

**Published:** 2017-12-12

**Authors:** Jian-Quan Luo, Huan Ren, Hoan Linh Banh, Mou-Ze Liu, Ping Xu, Ping-Fei Fang, Da-Xiong Xiang

**Affiliations:** ^1^Department of Pharmacy, The Second Xiangya Hospital, Central South University, Changsha, China; ^2^Institute of Clinical Pharmacy, Central South University, Changsha, China; ^3^Department of Clinical Pharmacology, Xiangya Hospital, Central South University, Changsha, China; ^4^Hunan Key Laboratory of Pharmacogenetics, Institute of Clinical Pharmacology, Central South University, Changsha, China; ^5^Department of Family Medicine, Faculty of Medicine and Dentistry, University of Alberta, Edmonton, AB, Canada

**Keywords:** coronary artery disease, type 2 diabetes mellitus, apolipoprotein E, epsilon2, epsilon3, epsilon4, genetic polymorphism

## Abstract

**Background and Objective:** Apolipoprotein E (APOE) plays important roles in lipoprotein metabolism and cardiovascular disease. Evidence suggests the *APOE* gene epsilon2/epsilon3/epsilon4 (ε2/ε3/ε4) polymorphisms might be associated with the susceptibility of coronary artery disease (CAD) in patients with type 2 diabetes mellitus (T2DM). However, no clear consensus has yet been established. Therefore, the aim of this meta-analysis is to provide a precise conclusion on the potential association between *APOE* ε2/ε3/ε4 polymorphisms and the risk of CAD in patients with T2DM based on case-control studies.

**Methods:** Pubmed, Embase, Chinese National Knowledge Infrastructure (CNKI), and Wanfang databases were searched for all relevant studies prior to August 2017 in English and Chinese language. The pooled odds ratios (ORs) and their corresponding 95% confidence intervals (CIs) were used to assess the strength of the relationships. The between-study heterogeneity was evaluated by Cochran's Q-test and the I^2^ index to adopt fixed- or random- effect models.

**Results:** A total of 13 studies were eligible for inclusion. There was evidence for significant associations between *APOE* ε4 mutation and the risk of CAD in patients with T2DM (for ε3/ε4 vs. ε3/ε3: OR = 1.69, 95% CI = 1.38–2.08, *P* < 0.001; for ε4/ε4 vs. ε3/ε3: OR = 2.72, 95% CI = 1.61–4.60, *P* < 0.001; for ε4/ε4+ε3/ε4 vs. ε3/ε3: OR = 1.83, 95% CI = 1.52–2.22, *P* < 0.001; for ε4 allele vs. ε3 allele: OR = 1.64, 95% CI = 1.40–1.94, *P* < 0.001). In contrast, no significant associations were found in genetic model of *APOE* ε2 mutation (for ε2/ε2 vs. ε3/ε3: OR = 1.67, 95% CI = 0.90–3.09, *P* = 0.104; for ε2/ε3 vs. ε3/ε3: OR = 1.18, 95% CI = 0.93–1.51, *P* = 0.175; for ε2/ε2+ε2/ε3 vs. ε3/ε3: OR = 1.26, 95% CI = 0.88–1.82, *P* = 0.212; for ε2 allele vs. ε3 allele: OR = 1.34, 95% CI = 0.98–1.84, *P* = 0.07).

**Conclusions:** The *APOE* gene ε4 mutation is associated with an increased risk of CAD in patients with T2DM, while the ε2 variation has null association with this disease.

## Introduction

Type 2 diabetes mellitus (T2DM) is a long-term metabolic disease with a high incidence and prevalence in the world. T2DM is often accompanied by various complications such as hypertension, dyslipidemia and coronary artery disease (CAD) (Naito and Miyauchi, 2017). As the disease progresses, patients with T2DM have a 2 to 4-fold increased risk for developing CAD compared with non-diabetic individuals (Mohan et al., 2001; Emerging Risk Factors et al., 2010). In addition, cardiovascular disease including CAD in patients with T2DM is associated with significant mortality (Zhang et al., 2014b; Freitas Lima et al., 2015). Therefore, early prevention and vigorous control of T2DM and its complications are becoming an ever-increasing global health priority. A better understanding of the etiology of CAD in patients with T2DM will result in better clinical management.

Dyslipidemia, hypertension, obesity, and smoking status are well-established risk factors for T2DM (Paneni et al., 2013; Wang et al., 2015a). Additionally, human genetic association studies have revealed that numerous genetic mutations and polymorphisms also play a critical role (Wei et al., 2014; Raj et al., 2015; Sumi et al., 2017). Among the previous studies, apolipoprotein E (APOE) gene has been regarded as one of the most likely candidate genes which may be associated with CAD in T2DM patients.

APOE is a class of plasma apolipoprotein totaling 299 amino acids, and it is involved in lipoprotein metabolism and the development of cardiovascular diseases (Zheng et al., 1998). The *APOE* gene is mapped to chromosome 19q13.2 in a cluster with apolipoprotein C1 and C2 gene, and it consists of three introns and four exons. *APOE* is a polymorphic gene and the most commonly studied alleles/isoforms are: epsilon2 (ε2), epsilon3 (ε3), and epsilon4 (ε4). The differences between the three isoforms are the location of 112 and 158 in the amino acid chain where cysteine or arginine is present. These three *APOE* alleles are determined by the rs7412 and rs429358 single-nucleotide polymorphisms. The three alleles, *APOE*-ε2 (cys112 and cys158), *APOE*-ε3 (cys112 and arg158) and *APOE*-ε4 (arg112 and arg158), yield six different genotypes for the *APOE* gene: ε2/ε2, ε2/ε3, ε2/ε4, ε3/ε3, ε3/ε4, and ε4/ε4. Because the ε3 allele or ε3/ε3 genotype is the most common allele or genotype among the population, they are well accepted as the “wild-type” and used as the “reference” in the genetic models (Zhang et al., 2000; Guo et al., 2007; Izar et al., 2009; Chaudhary et al., 2012; Hong et al., 2017).

The role of *APOE* ε2/ε3/ε4 polymorphisms in the development of CAD in patients with T2DM is widely studied, but the results are still controversial and conflicting. In 1998, Zheng et al. firstly investigated the association between *APOE* gene polymorphism and T2DM complicated with CAD in the Chinese population. The results showed that *APOE*-ε4 allele increased the risk of CAD in T2DM (Zheng et al., 1998). Other studies have also confirmed Zheng's findings (Chaaba et al., 2008; Hong et al., 2017). However, *APOE*-ε2 allele was also found to be associated with the risk of CAD in T2DM (Halim et al., 2012). In addition, no significant association between *APOE* ε2/ε3/ε4 polymorphisms and the risk of CAD in T2DM was reported in some studies (Zhang et al., 2000; Guo et al., 2007; Izar et al., 2009). To demonstrate with certainty the associations between the *APOE* ε2/ε3/ε4 polymorphisms and the risk of CAD in patients with T2DM, we conducted a systematic review and meta-analysis on published case-control studies.

## Materials and methods

### Literature search

This study was undertaken according to the methodology of MOOSE (Meta-analysis of Observational Studies in Epidemiology) statement (Stroup et al., 2000). We thoroughly searched all published studies in the Embase, PubMed, China National Knowledge Infrastructure (CNKI) and Wanfang databases up to August 2, 2017. The included articles were limited to Chinese and English language. The following keywords were used for searching: “apolipoprotein E” OR “APOE” AND “polymorphism” OR “single nucleotide polymorphism” OR “SNP” OR “variant” OR “variation” AND “coronary artery disease” OR “coronary heart disease” OR “CAD” OR “CHD” OR “atherosclerosis” OR “myocardial infarction” OR “myocardial infarct” OR “heart attack” OR “MI” AND “type 2 diabetes” OR “non-insulin dependent diabetes mellitus” OR “diabetes mellitus, type 2” OR “diabetes, type 2” OR “diabetes mellitus, non-insulin dependent” The Chinese databases were searched using the equivalent Chinese terms. In addition, hand searches for all related articles were performed. The detailed search strategies are presented in Supplementary Table 1.

### Inclusion and exclusion criteria

The first two investigators independently accessed the eligibility of the studies by screening the title, abstract and full-text, based on the inclusion and exclusion criteria. The inclusion criteria for all studies were as follows: (1) study on the associations between *APOE* ε2/ε3/ε4 polymorphisms and CAD in patients with T2DM, regardless of sample size. (2) case-control design. (3) detailed data for the *APOE* alleles or genotype distribution in case and control groups to estimate odds ratio (OR) with 95% confidence interval (CI). Exclusion criteria: (1) duplication of previous data; (2) review, comment and editorial; (3) no sufficient genotype data. Any dispute about the eligibility of an article was resolved by discussion.

### Data extraction

The data was drawn out based on a standard protocol. The following information was carefully extracted from all eligible publications independently by two authors (JQL and HR) using a standardized form: last name of first author, year of publication, study country, sample size in cases and controls, methods of genotyping, number genotypes and alleles. If similar data sets presented in different articles by the same research group, the data would be adopted only once. The collected data were compared, and possible disagreements were discussed by the authors and resolved with consensus.

### Quality score assessment

The study quality was independently assessed by two reviewers. Quality assessment of genetic associations between *APOE* ε2/ε3/ε4 polymorphisms and CAD in patients with T2DM is described in the Supplementary Table 2. The scores were adjusted according to the criteria developed for meta-analysis of molecular association studies by Thakkinstian et al. (2005). The total scores ranged from 0 to 13, with 13 representing the highest quality.

### Statistics analysis

All the statistical analysis in this study was performed using Stata 12.0 (StataCorp, College Station, TX). Hardy-Weinberg equilibrium was performed in control groups using the chi-square test. The combined OR and 95%CI were calculated to evaluate the strength of the association between the *APOE* ε2/ε3/ε4 polymorphisms and risk of CAD in T2DM subjects. The pooled ORs were, respectively, performed for nine genetic models (ε2/ε2 vs. ε3/ε3; ε2/ε3 vs. ε3/ε3; ε2/ε4 vs. ε3/ε3; ε3/ε4 vs. ε3/ε3; ε4/ε4 vs. ε3/ε3; ε2 allele vs. ε3 allele; ε4 allele vs. ε3 allele; ε2/ε2+ε2/ε3 vs. ε3/ε3; ε4/ε4+ε3/ε4 vs. ε3/ε3). The statistically significant level of the combined OR was determined by the *Z*-test with *P* < 0.05. Heterogeneity between studies was calculated by using the Cochran's Q-test and Higgins I^2^ index. In the absence of between-study heterogeneity (I^2^ < 50%), the fixed effect model (Mantel–Haenszel method) was chosen to calculate the pooled estimates. Otherwise, random effect model (DerSimonian and Laird method) would be adopted if the I^2^ > 50% (Higgins et al., 2003). Subgroup analysis was performed according to the source of patients (Chinese and non-Chinese). Galbraith plot analysis and sensitivity analysis were conducted to detect whether there were outliers that could be the potential sources of heterogeneity between studies when heterogeneity was moderately large. Publication bias was evaluated by Begg's funnel plot and Egger's regression test (Begg and Mazumdar, 1994; Egger et al., 1997). If there is evidence of significant publication bias, the trim and fill method was performed to assess the potential influence of publication bias (Duval and Tweedie, 2000).

## Results

### The characteristics of the included studies

**Figure 1:**
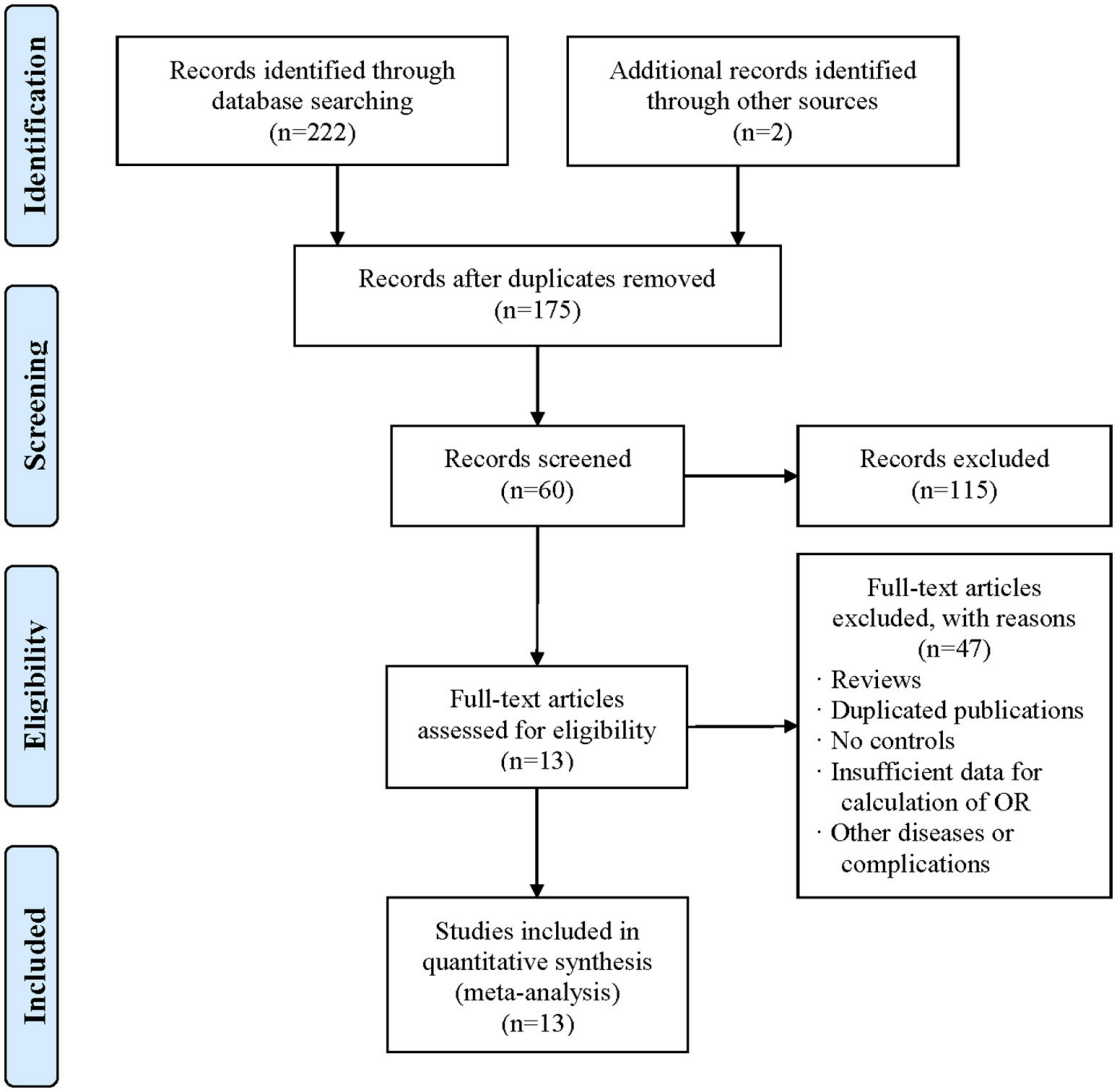
Flow diagram of study selection process. The term “n” in the boxes represens the number of corresponding studies.

As depicted in Figure 1, a total of 222 articles were obtained by online search, and 2 articles were included by manual search. After removing duplicates, 175 articles were included. After screening title and abstract, 115 articles were excluded. As a result, 13 articles (Zheng et al., 1998; Zhang et al., 2000, 2008; Pan et al., 2002; Guo et al., 2007; Chaaba et al., 2008; Izar et al., 2009; Shi et al., 2009; Vaisi-Raygani et al., 2010; Al-Majed et al., 2011; Chaudhary et al., 2012; Halim et al., 2012; Hong et al., 2017) were eligible for the meta-analysis. The characteristics of the included articles are summarized in Table 1. The included studies were conducted in several countries including China, Brazil, Thailand, Egypt, Iran, Kuwait, and Tunisia. All studies were performed in a case-control design and the sample sizes varied from 70 to 990. The quality score of the included studies ranged from 5 to 12 (mean: 9.69) out of a maximal score of 13.

**Table 1:** Characteristics of the included studies for this meta-analysis.

**First-author**	**Year**	**Country**	**Genotyping methods^a^**	**Quality score**	**Sample size**		**APOE genotypes distribution (case/control)**										
					**Case**	**Control**	**ε2/ε2**	**ε2/ε3**	**ε3/ε3**	**ε3/ε4**	**ε4/ε4**	**ε2/ε4**	**ε2**	**ε3**	**ε4**	**ε2/ε2+ ε2/ε3**	**ε3/ε4+ ε4/ε4**
Hong	2017	China	RT-PCR	10	114	106	1/1	14/11	61/72	31/20	2/0	5/2	21/15	167/175	40/22	15/12	33/20
Chaudhary	2012	Thailand	PCR-RFLP	12	147	155	1/1	11/2	88/117	46/30	1/4	0/1	13/5	233/266	48/39	12/3	47/34
Halim	2012	Egypt	PCR-RFLP	5	35	35	6/0	5/2	18/31	6/2	0/0	0/0	17/2	47/66	6/2	11/2	6/2
Al-Majed	2011	Kuwaiti	PCR-RFLP	9	41	105	3/7	1/2	21/73	4/6	12/15	0/2	7/18	47/154	28/38	4/9	16/21
Vaisi-Raygani	2010	Iran	PCR-RFLP	12	172	118	4/0	31/26	91/69	31/20	12/3	3/0	42/26	244/184	58/26	35/26	43/23
Shi	2009	China	PCR-RFLP	9	98	110	0/0	4/3	44/71	36/27	2/0	12/9	16/12	128/172	52/36	4/3	38/27
Izar	2009	Brazil	PCR-RFLP	11	386	604	3/7	60/86	241/388	57/81	9/4	14/31	80/131	599/943	89/120	63/93	66/85
Chaaba	2008	Tunisia	PCR-RFLP	9	71	86	0/0	3/9	57/68	NA	NA	0/1	NA	NA	NA	3/9	11/8
Zhang L	2008	China	PCR-RFLP	10	100	100	2/4	12/15	54/67	30/13	2/1	0/0	16/23	150/162	34/15	14/19	32/14
Guo	2007	China	Multi-ARMS-PCR	11	40	40	0/0	2/1	22/29	13/7	1/0	2/3	4/4	59/66	17/10	2/1	14/7
Pan	2002	China	PCR-RFLP	11	24	63	0/1	4/7	12/45	6/8	0/0	2/2	6/11	34/105	8/10	4/8	6/8
Zhang WH	2000	China	PCR-RFLP	9	61	63	1/0	2/7	46/50	10/6	1/0	1/0	5/7	104/113	13/6	3/7	11/6
Zheng	1998	China	PCR-RFLP	8	33	78	NA	NA	22/59	NA	NA	NA	NA	NA	NA	3/15	8/4

a *Multi-ARMS-PCR: multiplex amplification refractory mutation system-polymerase chain reaction; PCR-RFLP: polymerase chain reaction restriction fragment length polymorphism; RT-PCR: real-time polymerase chain reaction*.

*NA: not available*.

### Quantitative synthesis

**Figure 2:**
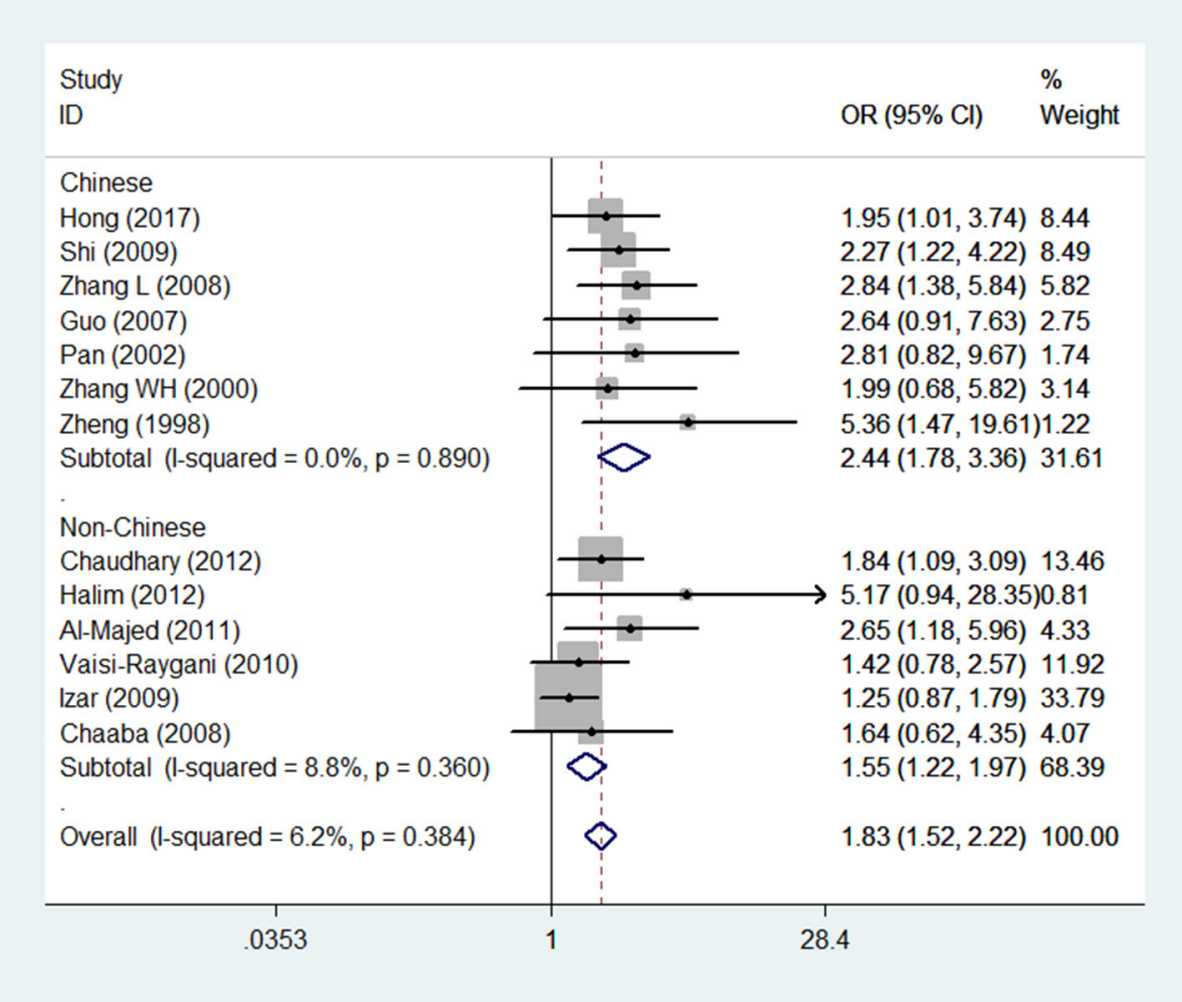
Forest plot for the association between *APOE* gene polymorphism and the risk of coronary artery diseases in type 2 diabetes patients under the genetic model of ε4/ε4+ε3/ε4 vs. ε3/ε3. The center of each square represents the OR, the area of the square is for the weight of studies, and the horizontal line indicates the 95% CI.

**Figure 3:**
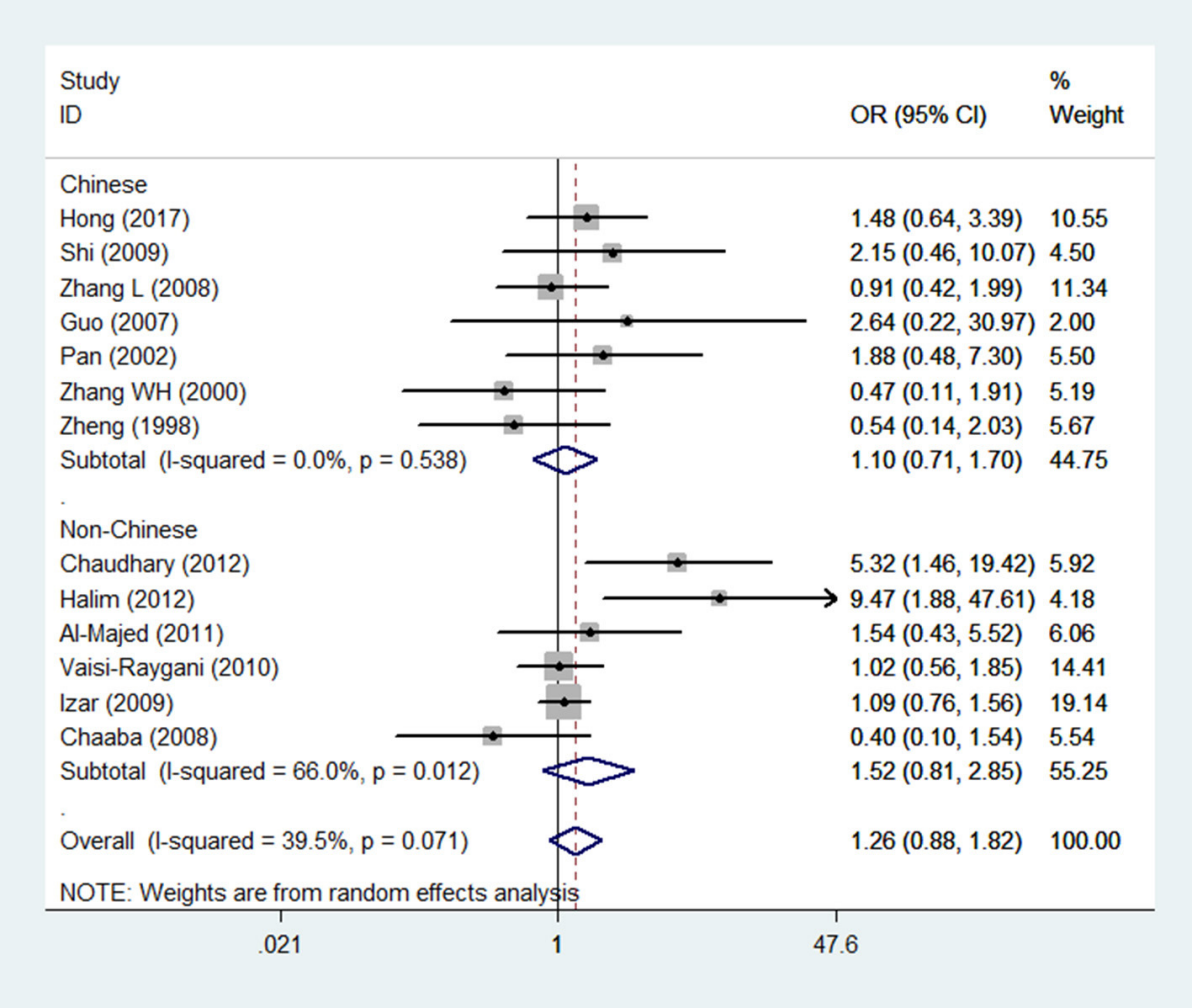
Forest plot for the association between *APOE* gene polymorphism and the risk of coronary artery diseases in type 2 diabetes patients under the genetic model of ε2/ε2+ε2/ε3 vs. ε3/ε3. The center of each square represents the OR, the area of the square is for the weight of studies, and the horizontal line indicates the 95% CI.

The main results of this meta-analysis for the association between *APOE* ε2/ε3/ε4 polymorphisms and the risk of CAD in T2DM patients are presented in Table 2. There was significant association in three genetic models which demonstrate, ε4 mutation contributed to an increased risk of CAD in patients with T2DM (Figure 2). The pooled results for the three genetic models in the overall analysis were as follows: for ε3/ε4 vs. ε3/ε3: OR = 1.69, 95% CI = 1.38–2.08, *P* < 0.001; for ε4/ε4 vs. ε3/ε3: OR = 2.72, 95% CI = 1.61–4.60, *P* < 0.001; for ε4/ε4+ε3/ε4 vs. ε3/ε3: OR = 1.83, 95% CI = 1.52–2.22, *P* < 0.001. In contrast, the ε2 variation had null association with this disease (Figure 3). No significant association in the overall analysis was found in genetic model of ε2/ε2 vs. ε3/ε3 (OR = 1.67, 95% CI = 0.90–3.09, *P* = 0.104); ε2/ε3 vs. ε3/ε3 (OR = 1.18, 95% CI = 0.93–1.51, *P* = 0.175); ε2/ε4 vs. ε3/ε3 (OR = 1.20, 95% CI = 0.78–1.84, *P* = 0.405); ε2/ε2+ε2/ε3 vs. ε3/ε3 (OR = 1.26, 95% CI = 0.88–1.82, *P* = 0.212). In addition, the genetic models of allele-based contrasts in the overall analysis also revealed a statistically significant pooled estimates for ε4 allele vs. ε3 allele (OR = 1.64, 95% CI = 1.40–1.94, *P* < 0.001) but not for ε2 allele vs. ε3 allele (OR = 1.34, 95% CI = 0.98–1.84, *P* = 0.07).

**Table 2:** Meta-analysis results of the associations between *APOE* ε2/ε3/ε4 polymorphisms and risk of coronary artery diseases in type 2 diabetes patients.

**Genetic model**	**Pooled OR (95%CI)**	***Z*-value**	***P*_meta−analysis_**	**NO. of studies**	**Model^a^**	***P*_heterogeneity_^b^**	**I^2^%**
ε2/ε2 vs. ε3/ε3	1.67(0.90–3.09)	1.62	0.104	9	F	0.532	0.00
Chinese	2.03(0.98–4.21)	0.02	0.984	4	F	0.841	0.00
Non-Chinese	1.01(0.31–3.32)	1.90	0.058	5	F	0.208	32.00
ε2/ε3 vs. ε3/ε3	1.18(0.93–1.51)	1.36	0.175	12	F	0.151	30.10
Chinese	1.21(0.76–1.95)	0.80	0.423	6	F	0.450	0.00
Non-Chinese	1.32(0.72–2.42)	0.89	0.374	6	R	0.053	54.30
ε2/ε4 vs. ε3/ε3	1.20(0.78–1.84)	0.83	0.405	10	F	0.493	0.00
Chinese	2.17(1.10–4.28)	2.22	0.026	5	F	0.852	0.00
Non-Chinese	0.79(0.44–1.41)	0.79	0.428	5	F	0.746	0.00
ε3/ε4 vs. ε3/ε3	1.69(1.38–2.08)	4.99	<0.001	11	F	0.312	13.90
Chinese	2.22(1.59–3.09)	4.71	<0.001	6	F	0.954	0.00
Non-Chinese	1.42(1.09–1.85)	2.57	0.010	5	F	0.186	35.30
ε4/ε4 vs. ε3/ε3	2.72(1.61–4.60)	3.72	<0.001	9	F	0.807	0.00
Chinese	4.26(1.16–15.61)	2.18	0.029	5	F	0.980	0.00
Non-Chinese	2.45(1.37–4.37)	3.03	0.002	4	F	0.291	19.70
ε2/ε2+ε2/ε3 vs. ε3/ε3	1.26(0.88–1.82)	1.25	0.212	13	R	0.071	39.50
Chinese	1.08(0.71–1.65)	0.34	0.734	7	F	0.538	0.00
Non-Chinese	1.52(0.81–2.85)	1.30	0.193	6	R	0.012	66.00
ε4/ε4+ε3/ε4 vs. ε3/ε3	1.83(1.52–2.22)	6.24	<0.001	13	F	0.384	6.20
Chinese	2.44(1.78–3.36)	5.51	<0.001	7	F	0.890	0.00
Non-Chinese	1.55(1.22–1.97)	3.60	<0.001	6	F	0.360	8.80
ε2 allele vs. ε3 allele	1.34(0.98–1.84)	1.81	0.070	11	R	0.054	44.70
Chinese	1.19(0.84–1.69)	0.99	0.324	6	F	0.536	0.00
Non-Chinese	1.67(0.93–3.03)	1.71	0.088	5	R	0.007	71.50
ε4 allele vs. ε3 allele	1.64(1.40–1.94)	5.97	<0.001	11	F	0.284	16.80
Chinese	2.08(1.58–2.74)	5.21	<0.001	6	F	0.987	0.00
Non-Chinese	1.44(1.17–1.77)	3.50	<0.001	5	F	0.138	42.60

*OR, odd ratio; CI, confidence interval*.

a *F: fixed random effect model; R: random effect model*.

b *P_heterogeneity_ value for between-study heterogeneity based on Cochran's Q test*.

In the subgroup analysis according to the source of patients (Chinese and non-Chinese), the pooled ORs of all genetic models except the ε2/ε4 vs. ε3/ε3 model were consistent with the results in the overall population. In the Chinese population, the ε2/ε4 genotype increased the risk of CAD in patients with T2DM (OR = 2.17, 95% CI = 1.10–4.28, *P* = 0.026).

### Heterogeneity analysis

As shown in Table 2, there was moderate between-study heterogeneity in the genetic model of ε2 allele vs. ε3 allele (*P*_heterogeneity_ = 0.054, I^2^ = 44.70%) and ε2/ε2+ε2/ε3 vs. ε3/ε3 (*P*_heterogeneity_ = 0.071, I^2^ = 39.50%) in the overall analysis. However, no significant heterogeneity was found in other genetic models (for ε2/ε2 vs. ε3/ε3: *P*_heterogeneity_ = 0.532, I^2^ = 0%; for ε2/ε3 vs. ε3/ε3: *P*_heterogeneity_ = 0.151, I^2^ = 30.10%; for ε2/ε4 vs. ε3/ε3: *P*_heterogeneity_ = 0.493, I^2^ = 0%; for ε3/ε4 vs. ε3/ε3: *P*_heterogeneity_ = 0.312, I^2^ = 13.90%; for ε4/ε4 vs. ε3/ε3: *P*_heterogeneity_ = 0.807, I^2^ = 0%; for ε4 allele vs. ε3 allele: *P*_heterogeneity_ = 0.284, I^2^ = 16.80%; for ε4/ε4+ε3/ε4 vs. ε3/ε3: *P*_heterogeneity_ = 0.384, I^2^ = 6.20%). The heterogeneity analysis results indicated that the pooled results of this meta-analysis in most genetic models were statistically steady and robust. In addition, subgroup analysis indicated that there was no heterogeneity under all nine genetic models in the Chinese population.

### Galbraith plot analysis and sensitivity analysis

**Figure 4:**
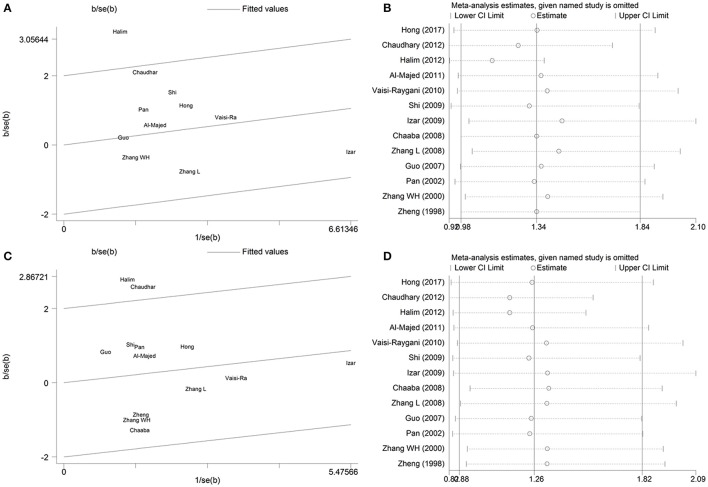
Galbraith plot analysis and sensitivity analysis of the association between *APOE* gene polymorphism and the risk of coronary artery diseases in type 2 diabetes patients under the genetic model of ε2 allele vs. ε3 allele **(A,B)** and ε2/ε2+ε2/ε3 vs. ε3/ε3 **(C,D)**. For sensitivity analysis, open circle indicates the pooled ORs, horizontal lines represent the 95% CIs, given named study is omitted.

There was evidence of moderately large between-study heterogeneity in the genetic model of ε2 allele vs. ε3 allele (*P*_heterogeneity_ = 0.054, I^2^ = 44.70%) and ε2/ε2+ε2/ε3 vs. ε3/ε3 (*P*_heterogeneity_ = 0.071, I^2^ = 39.50%), so Galbraith plot analysis and sensitivity analysis were performed to detect the possible sources of heterogeneity. Under the genetic model of ε2 allele vs. ε3 allele, the Galbraith plot analysis (Figure 4A) showed that the Halim et al. study was the outlier, which is consistent with the results of sensitivity analysis (Figure 4B). No heterogeneity existed after this outlier study was omitted (*P*_heterogeneity_ = 0.460, I^2^ = 0%). Thus, the study by Halim et al. may be the source of heterogeneity in the meta-analysis for the ε2 allele vs. ε3 genetic model.

Similarly, under the genetic model of ε2/ε2+ε2/ε3 vs. ε3/ε3, the Galbraith plot analysis (Figure 4C) and sensitivity analysis (Figure 4D) indicated that Halim and Chaudhary's study were the outliers. When the two outlier studies were omitted, no heterogeneity existed in the remaining studies (*P*_heterogeneity_ = 0.681, I^2^ = 0%). Therefore, the studies of Halim et al. and Chaudhary et al. may be the main contributors to the source of heterogeneity in the meta-analysis for the ε2/ε2+ε2/ε3 vs. ε3/ε3 genetic model.

### Publication bias

**Figure 5:**
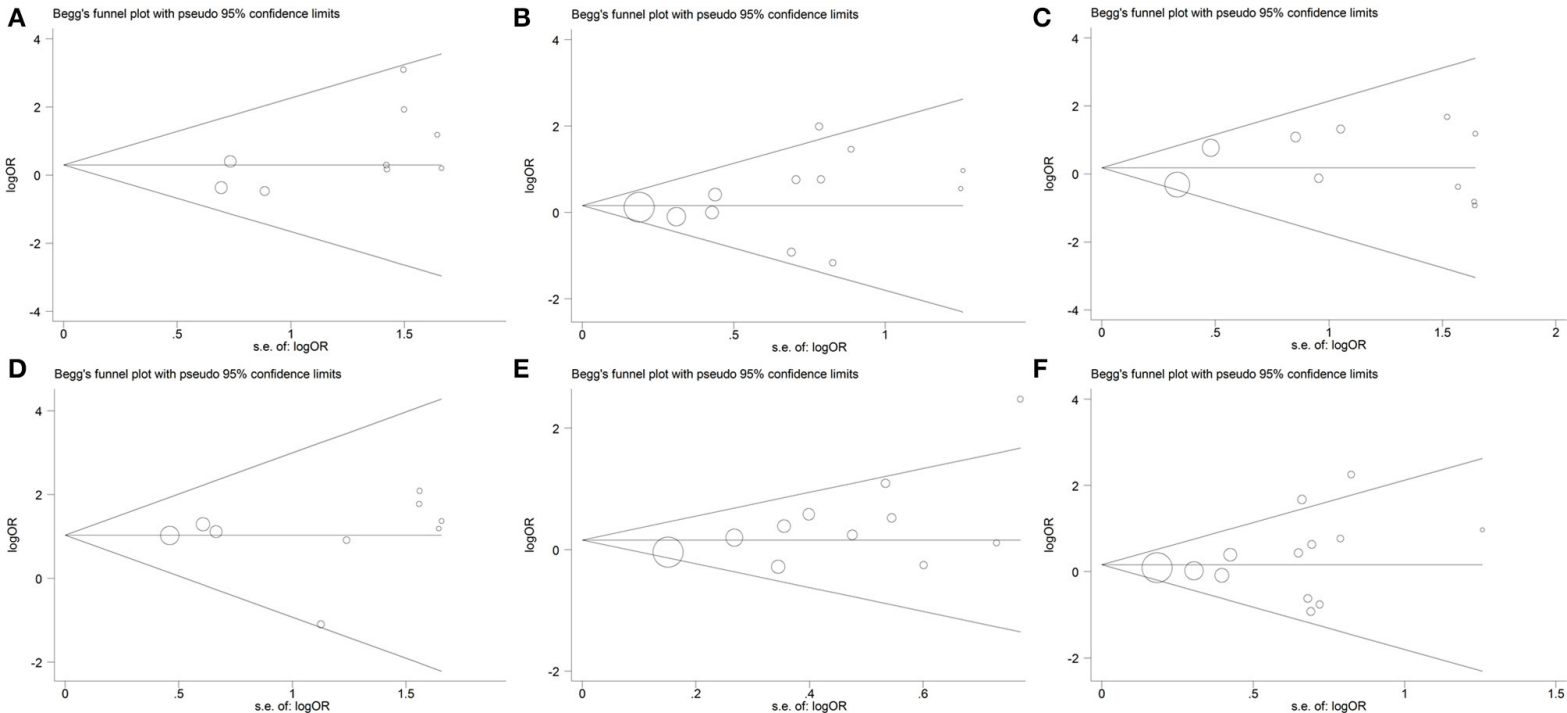
Begg's funnel plot for the association between *APOE* gene polymorphism and the risk of coronary artery diseases in type 2 diabetes patients under the genetic model of ε2/ε2 vs. ε3/ε3 **(A)**, ε2/ε3 vs. ε3/ε3 **(B)**, ε2/ε4 vs. ε3/ε3 **(C)**, ε4/ε4 vs. ε3/ε3 **(D)**, ε2 allele vs. ε3 allele **(E)**, and ε2/ε2+ε2/ε3 vs. ε3/ε3 **(F)**. Size of the open circles is proportional to the weight of studies.

No obvious asymmetry was observed in the shape of the funnel plot for the following genetic models: ε2/ε2 vs. ε3/ε3 (Figure 5A); ε2/ε3 vs. ε3/ε3 (Figure 5B); ε2/ε4 vs. ε3/ε3 (Figure 5C); ε4/ε4 vs. ε3/ε3 (Figure 5D); ε2 allele vs. ε3 allele (Figure 5E); ε2/ε2+ε2/ε3 vs. ε3/ε3 (Figure 5F). In addition, the Begg's test and Egger's test also did not show any evidence of publication bias (*P*_Begg_ = 0.251 and *P*_Egger_ = 0.08 for ε2/ε2 vs. ε3/ε3, *P*_Begg_ = 0.373 and *P*_Egger_ = 0.320 for ε2/ε3 vs. ε3/ε3, *P*_Begg_ = 0.283 and *P*_Egger_ = 0.403 for ε2/ε4 vs. ε3/ε3, *P*_Begg_ = 0.466 and *P*_Egger_ = 0.988 for ε4/ε4 vs. ε3/ε3, *P*_Begg_ = 0.119 and *P*_Egger_ = 0.053 for ε2 allele vs. ε3 allele, *P*_Begg_ = 0.300 and *P*_Egger_ = 0.331 for ε2/ε2+ε2/ε3 vs. ε3/ε3).

**Figure 6:**
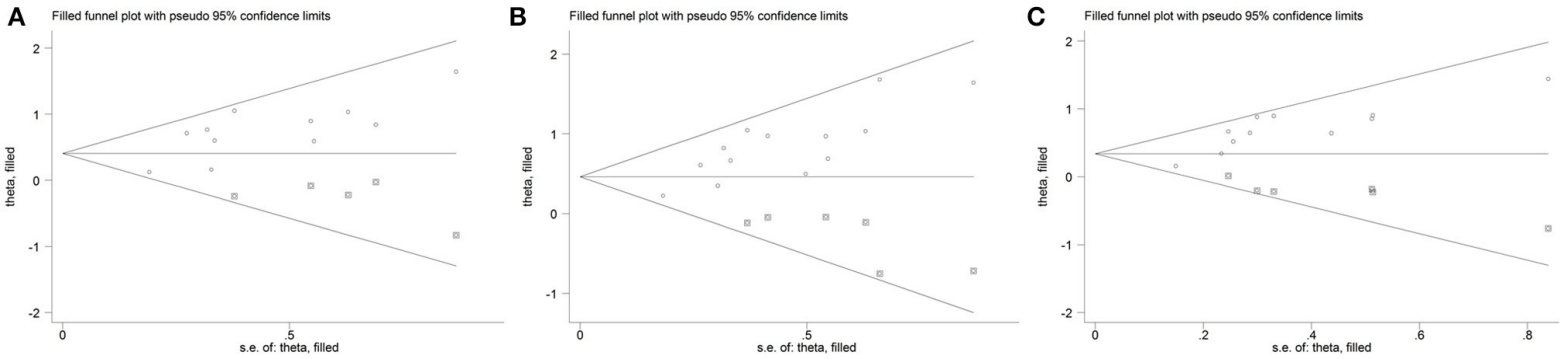
Funnel plot with trim and fill method for the association between *APOE* gene polymorphism and the risk of coronary artery diseases in type 2 diabetes patients under the genetic model of ε3/ε4 vs. ε3/ε3 **(A)**, ε4/ε4+ε3/ε4 vs. ε3/ε3 **(B)**, and ε4 allele vs. ε3 allele **(C)**. Circle represents the included studies; Square represents the possibly missing studies.

The results from the following three genetic models ε3/ε4 vs. ε3/ε3; ε3/ε4+ε4/ε4 vs. ε3/ε3 and ε4 allele vs. ε3 allele performed by Begg's test (*P*_Begg_ = 0.213, *P*_Begg_ = 0.033, and *P*_Begg_ = 0.043, respectively) or Egger's test (*P*_Egger_ = 0.013; *P*_Egger_ = 0.001 and *P*_Egger_ = 0.001, respectively) revealed publication bias. Nevertheless, by using the trim and fill method, the recalculated estimates (OR = 1.50, 95%CI = 1.24–1.82; OR = 1.59, 95%CI = 1.34–1.89 and OR = 1.40, 95%CI = 1.22–1.62, respectively) remained statistically significant, which indicated that our meta-analysis results were steady and not influenced by publication bias. Figure 6 shows the funnel plot of trim and fill method in the genetic model of ε3/ε4 vs. ε3/ε3 (Figure 6A), ε3/ε4+ε4/ε4 vs. ε3/ε3 (Figure 6B), ε4 allele vs. ε3 allele (Figure 6C).

## Discussion

T2DM is a well-established risk factor for the development of CAD. The management of CAD in patients with T2DM poses great challenges to the medical profession (Wei et al., 2015). The identification of susceptibility genes would be very helpful for the management of CAD in patients with T2DM. The link between *APOE* ε2/ε3/ε4 polymorphisms and CAD in diabetic patients has been highlighted in our study. This meta-analysis provides evidence for the significant associations between *APOE* ε4 mutation (ε3/ε4 vs. ε3/ε3; ε4/ε4 vs. ε3/ε3; ε4/ε4+ε3/ε4 vs. ε3/ε3; ε4 allele vs. ε3 allele) and an elevated risk of CAD in patients with T2DM. In contrast, no significant association was found in genetic model of *APOE* ε2 variation (ε2/ε2 vs. ε3/ε3; ε2/ε3 vs. ε3/ε3; ε2/ε2+ε2/ε3 vs. ε3/ε3; ε2 allele vs. ε3 allele). However, CAD in patients with T2DM is believed to be multifactorial and involved in many susceptibility genes with small individual effects. Therefore, the integration of information derived from several polymorphisms in multiple susceptibility genes may become clinically useful.

It has been reported that lipoprotein-related mechanisms are associated with the impairment of the cardiovascular system among patients with diabetes (Jenkins et al., 2004). For example, serum low-density lipoprotein cholesterol (LDL-C) level was identified as an independent risk factor for CAD in T2DM patients (Jayashankar et al., 2016). APOE is initially recognized for its important role in plasma lipid metabolism and thus affects the serum lipid profiles in the body. The three *APOE* alleles (ε2, ε3, ε4) differ from each other by only one or two amino acids at positions 112 and 158, but these slight differences alter the structure and function of APOE. In general, the *APOE*-ε4 allele is associated with higher and the *APOE*-ε2 allele with lower total plasma cholesterol and LDL-C concentrations compared with the *APOE*-ε3 allele (Bennet et al., 2007; Larifla et al., 2017). Therefore, abnormalities of lipoprotein metabolism may explain, at least in part, the associations between *APOE* ε2/ε3/ε4 polymorphisms and the risk of CAD in patients with T2DM.

Several meta-analysis studies have been conducted to assess the association between *APOE* ε2/ε3/ε4 polymorphisms and risk of CAD in the general population. In 2004, Song et al firstly found that carriers of the *APOE*-ε4 allele had a 42% increased risk for CAD (OR = 1.42, 95% CI = 1.26–1.61) compared with the ε3/ε3 genotypes (Song et al., 2004). Xu et al. found similar results which showed that the ε4 allele had a 46% higher risk of CAD (OR = 1.46, 95% CI = 1.28–1.66) (Xu et al., 2016). Similar findings were also observed in other meta-analysis (Yin et al., 2013; Xu et al., 2014, 2016; Zhang et al., 2014a, 2015; Wang et al., 2015b). Interestingly, the role of *APOE*-ε2 allele in the risk of CAD may be dependent on the patient ethnicity (Xu et al., 2016). In addition, the association between *APOE* ε2/ε3/ε4 polymorphisms and the risk of T2DM in the general population was also well explored in previous meta-analysis (Anthopoulos et al., 2010; Yin et al., 2014). The results indicated that both *APOE* ε2 and ε4 alleles were associated with an increased risk of T2DM in the general population. In 2015, Wu et al. performed a meta-analysis on the association between *APOE* ε2/ε3/ε4 polymorphisms and T2DM patients with CAD among Chinese Han population. They found that *APOE*-ε4 allele resulted in an increased risk of T2DM patients with CAD in China (Wu et al., 2015). However, only five individual studies were included in their meta-analysis. To our knowledge, our meta-analysis represents the largest study to investigate the association between *APOE* ε2/ε3/ε4 polymorphisms and risk of CAD in the T2DM patients.

Heterogeneity across studies is common in meta-analysis of genetic association study (Munafo and Flint, 2004). Heterogeneity should be taken into consideration in the interpretation of the meta-analysis results. However, one of the strengths in this meta-analysis was the lack of significant heterogeneity in all genetic models except the genetic model of ε2 allele vs. ε3 allele. Between-study heterogeneity can be attributed to the potential differences such as the definition of disease, ethnicity, genotyping methods and sample size in the included studies. To explore the potential sources of heterogeneity under the genetic model of ε2 allele vs. ε3 allele, Galbraith plot analysis and sensitivity analysis were employed to detect whether there were outliers that could be the potential sources of heterogeneity between studies. The study conducted by Halim et al was considered as the main contributors to between-study heterogeneity. The heterogeneity was effectively decreased after omitting the study. The frequency of *APOE*-ε3 allele was nearly 95% in Halim's study, whereas lower than 90% in other studies (Zhang et al., 2008; Izar et al., 2009; Chaudhary et al., 2012; Hong et al., 2017). Consequently, the heterogeneity can be due to the distinct frequency of *APOE* ε2/ε3/ε4 polymorphisms among the included studies. Although Halim's study caused the substantial heterogeneity in the genetic model of ε2 allele vs. ε3 allele, the pooled effect was still insignificant after removing it.

There are several limitations in this meta-analysis that should be noted. First, the included studies were limited to only English or Chinese languages in our research and some eligible studies may be published in other languages, which would cause bias of the results. Second, all the included studies in this meta-analysis were the type of retrospective case-control studies, which may result in some selection bias. Third, publication bias existed in the following three genetic models: ε3/ε4 vs. ε3/ε3; ε3/ε4+ε4/ε4 vs. ε3/ε3; ε4 allele vs. ε3 allele. However, by using the trim and fill method, the recalculated ORs and their 95% CIs did not change, which indicated the stability and robustness of meta-analysis results. Last but not the least, T2DM complicated with CAD is a multifactorial disease caused by both genetic and environmental factors. The *APOE*-environment interactions should be considered. For example, the study by Talmud et al. has found that the impact of the *APOE*-ε4 on the risk of CAD appeared to be restricted to smokers (Talmud et al., 2004).

In conclusion, we observed a significant association between the *APOE* gene ε4 mutation and an increased risk of CAD in patients with T2DM, while the ε2 variation had null association with this disease. Taking into account the above limitations, more studies with larger sample size and incorporated with gene-environment interactions are needed to definitively determine the association between the *APOE* gene ε2/ε3/ε4 polymorphisms and the risk of CAD in patients with T2DM.

## Author contributions

Conceived and designed the study: J-QL and HR. Performed the search: J-QL, HR, M-ZL. Analyzed the data: J-QL and HR. Contributed reagents/material/analysis tools: J-QL, HR, M-ZL, PX, P-FF, and D-XX. Wrote and review the manuscript: J-QL, HR and HB. Reference collection, data management, statistical analyses, paper writing, and study design: J-QL and HR.

### Conflict of interest statement

The authors declare that the research was conducted in the absence of any commercial or financial relationships that could be construed as a potential conflict of interest.
