# Supplementary material for: The Associations between Apolipoprotein E Gene Epsilon2/Epsilon3/Epsilon4 Polymorphisms and the Risk of Coronary Artery Disease in Patients with Type 2 Diabetes Mellitus
Source: Front Physiol. 2017 Dec 12;8:1031. doi: 10.3389/fphys.2017.01031 (PMC5732920; doi:10.3389/fphys.2017.01031)
Supplement: Supplementary file 1 [file Table1.DOCX]

Supplementary Table 1 The detailed search strategies of the association between *APOE* gene polymorphism and the risk of coronary artery diseases in patients with type 2 diabetes mellitus

| Database | Search strategy | |
| --- | --- | --- |
| Pubmed | #1 | "Diabetes Mellitus, Type 2"[Mesh] |
|  | #2 | Noninsulin Dependent Diabetes Mellitus |
|  | #3 | Diabetes, Type 2 |
|  | #4 | Type 2 Diabetes |
|  | #5 | Diabetes Mellitus, Noninsulin Dependent |
|  | #6 | #1 OR #2 OR #3 OR #4 OR #5 |
|  | #7 | "Coronary Artery Disease"[Mesh] |
|  | #8 | coronary heart disease |
|  | #9 | CAD |
|  | #10 | CHD |
|  | #11 | Atherosclerosis |
|  | #12 | "Myocardial Infarction"[Mesh] |
|  | #13 | Myocardial Infarct |
|  | #14 | Heart Attack |
|  | #15 | MI |
|  | #16 | #7 OR #8 OR #9 OR #10 OR #11 OR #12 OR #13 OR #14 OR #15 |
|  | #17 | "Apolipoproteins E"[Mesh] |
|  | #18 | ApoE |
|  | #19 | #17 OR #18 |
|  | #20 | "Mutation"[Mesh] |
|  | #21 | polymorphism |
|  | #22 | Single Nucleotide Polymorphism |
|  | #23 | SNP |
|  | #24 | variant |
|  | #25 | variation |
|  | #26 | #20 OR #21 OR #22 OR #23 OR #24 OR #25 |
|  | #27 | #6 AND #16 AND #19 AND #26 |
|  | | |
| Embase | #1 | type 2 diabetes'/exp |
|  | #2 | non insulin dependent diabetes mellitus' |
|  | #3 | diabetes mellitus, type 2' |
|  | #4 | diabetes, type 2' |
|  | #5 | diabetes mellitus, noninsulin dependent' |
|  | #6 | #1 OR #2 OR #3 OR #4 OR #5 |
|  | #7 | coronary artery disease'/exp |
|  | #8 | coronary heart disease' |
|  | #9 | cad |
|  | #10 | chd |
|  | #11 | atherosclerosis |
|  | #12 | myocardial infarction' |
|  | #13 | myocardial infarct' |
|  | #14 | heart attack' |
|  | #15 | mi |
|  | #16 | #7 OR #8 OR #9 OR #10 OR #11 OR #12 OR #13 OR #14 OR #15 |
|  | #17 | apolipoprotein e'/exp |
|  | #18 | apoe |
|  | #19 | #17 OR #18 |
|  | #20 | polymorphism |
|  | #21 | single nucleotide polymorphism' |
|  | #22 | snp |
|  | #23 | variant |
|  | #24 | variation |
|  | #25 | #20 OR #21 OR #22 OR #23 OR #24 |
|  | #26 | #6 AND #16 AND #19 AND #25 |
