# Supplementary material for: The Associations between Apolipoprotein E Gene Epsilon2/Epsilon3/Epsilon4 Polymorphisms and the Risk of Coronary Artery Disease in Patients with Type 2 Diabetes Mellitus
Source: Front Physiol. 2017 Dec 12;8:1031. doi: 10.3389/fphys.2017.01031 (PMC5732920; doi:10.3389/fphys.2017.01031)
Supplement: Supplementary file 2 [file Table2.DOC]

Supplementary Table 2 Criteria for quality assessment of genetic association between ApoE e2/e3/e4 polymorphisms and coronary artery disease in patients with type 2 diabetes mellitus

| Criteria | Quality score |
| --- | --- |
| Representativeness of cases |  |
| A. Consecutive/randomly selected from case population with clearly defined random frame | 2 |
| B. Consecutive/randomly selected from case population without clearly defined random frame or with extensive inclusion criteria | 1 |
| C. Method of selection not described | 0 |
| Representativeness of controls |  |
| D. Controls were consecutive/randomly drawn from the same area (ward/community) as cases with the same criteria | 2 |
| E. Controls were consecutive/randomly drawn from a different area than cases | 1 |
| F. Not described | 0 |
| Ascertainment of cases |  |
| G. Clearly described objective criteria for diagnosis of coronary artery disease and type 2 diabetes mellitus | 2 |
| H. Diagnosis of coronary artery disease and type 2 diabetes mellitus by patient self-report or by patient history | 1 |
| I. Not described | 0 |
| Ascertainment of controls |  |
| J. Clinical examinations were performed on controls to prove that controls did not have coronary artery disease and type 2 diabetes mellitus | 2 |
| K. Article merely stated that controls were subjects who did not have coronary artery disease and type 2 diabetes mellitus; no proof provided | 1 |
| L. Not described | 0 |
| Ascertainment of genotyping examination |  |
| M. Genotyping done under ‘‘blind’’ conditions | 1 |
| N. Unblended or not mentioned | 0 |
| Test for Hardy–Weinberg equilibrium |  |
| O. Hardy–Weinberg equilibrium in control group | 2 |
| P. Hardy–Weinberg disequilibrium in control group | 1 |
| Q. Hardy–Weinberg equilibrium not checked | 0 |
| Association assessment |  |
| R. Assessed association between genotypes and coronary artery disease in type 2 diabetic patients with appropriate statistic and adjusting confounders | 2 |
| S. Assessed association between genotypes and coronary artery disease in type 2 diabetic patients with appropriate statistic without adjusting confounders | 1 |
| T. Inappropriate statistic used | 0 |
